# A Case of Chronic Sulphur Poisoning

**Published:** 1888-06

**Authors:** 


### A CASE OF CHRONIC SULPHUR POISONING.

(Dr. Eichbaum, Leipzig, in *Berl. Klin. Wochenschrift*, 42, 1887.)

R., thirty-seven years old, healthy, not weighed down by neuropathic heredity, well developed and well nourished, complains for some time that he can neither stand nor walk without reeling. The color of his face is pale and sallow; forehead covered with sweat; the pupils, wide, staring, do not react to light, nor to cutaneous irritation; mobility of eye natural; tongue is put out straight, trembles somewhat, and is heavy coated; not a trace of paralysis, neither in face nor extremities; sounds of the heart dull but regular, no enlargement; lungs and other organs normal; pulse 124, very small; temperature of body rather diminished; head is carried rather backward and to the right, and the muscles of the right side of the neck feel hard and tense, especially the superficial ones; occipital headache, nausea, and oppression of chest; sensitive to least pressure in gastric region; headache was his complaint for years, exacerbating at irregular intervals; always with sensation of vertigo, nausea; spasmodic stiffness, followed by sensation of weakness of the muscles of the right side of the neck; bowels move rather frequently.

Patient has used for the last two years, on account of excessive dandruff, a pomade of vasaline 100, wax 5, flor. sulphur 10, ol. rosarum a few drops. This pomade was used every other day. Under the action of fat and heat the sulphur is decomposed, sulphuretted hydrogen entering the body through the scalp and by breathing, and the whole known symptoms of its chronic poisoning are headache, vertigo, paleness of face, frequent weak pulse, nausea, colic, and diarrhœa. The sulphuretted hydrogen acts on the blood by the central nervous system. Its action on the latter also explains the clonic-tonic spasms of the muscles of the right side of the neck, and the dilatation and loss of reaction of the pupil. Hot baths, fresh air, and removal of the cause soon relieved him of all his complaints.

The case verifies again every symptom of the patient and found in the provings of this great polycrest. Thus Allen, IX, gives: 129, long continued giddy confusion of the whole head; 154, vertigo while walking, like reeling; 158, vertigo while walking in the open air (after supper), she did not dare to stoop nor to look down; 392, weight and hot feeling in occiput; 404, drawing, aching pain in the occiput to the nape, at night pressive pain in occiput; 410, pulsating in left side of occiput, that at last changes to a jerking; 420, for many years scaliness of scalp, so that a large quantity covered his clothing. After taking Sulphur: 630, remarkable dilatation of pupils, distortion of left pupil; 850, complexion showed a remarkable change—it had a dirty, earthy appearance; 1020, coated tongue, thickly furred; 1039, convulsive shooting in tip of tongue; 1061, mouth dry, insipid, and sticky; 1377, nausea every morning, to faintness; 1441, epigastric region extremely painful to touch; 1622, rumbling and gurgling in abdomen; 1701, griping in bowels, followed soon by a liquid motion, whereupon the pain ceased; 1747, colic before every stool; 2410, oppression of chest, with inclination to breathe deeply; 2598, bruised pain in the muscles of the spine, pressive pain in the back beneath the scapulæ; 2594, pain as from a sprain in the right scapula; 2598, a very large symptom, like that of R., closing; close to the right scapula an aching, tensive pain, as if the muscles were too short.

Dear Doctor! Every verification has its own value, and here every symptom of the patient is verified in the proving, upholding fully the great value of our law, without going deeply into chemical abstractions, although many authors affirm that the action of Sulphur consists in its decomposition to sulphuretted hydrogen, for our provings give us fetid flatulence and diarrhœac stools smelling like rotten eggs. But how is it with the decomposition when a high potency of Sulphur (microscopically a myth) removes the symptoms in the body and from the body, which in the proving was caused by a low potency, or the mother tincture, or even by the crude drug? S. L.

